# Small molecule inhibitors of fungal Δ(9) fatty acid desaturase as antifungal agents against *Candida auris*

**DOI:** 10.3389/fcimb.2024.1434939

**Published:** 2024-08-30

**Authors:** Faiza Tebbji, Anagha C. T. Menon, Inès Khemiri, Daniel J. St-Cyr, Louis Villeneuve, Antony T. Vincent, Adnane Sellam

**Affiliations:** ^1^ Montreal Heart Institute/Institut de Cardiologie de Montréal, Université de Montréal, Montreal, QC, Canada; ^2^ Department of Microbiology, Infectious Diseases and Immunology, Faculty of Medicine, Université de Montréal, Montreal, QC, Canada; ^3^ Institute for Research in Immunology and Cancer (IRIC), Department of Medicine, Université de Montréal, Montreal, QC, Canada; ^4^ Department of Animal Sciences, Université Laval, Quebec City, QC, Canada; ^5^ Institute of Integrative and Systems Biology, Université Laval, Quebec City, QC, Canada

**Keywords:** aryl-carbohydrazide, antifungal, *Candida auris*, delta 9 fatty acid desaturase, unsaturated fatty acids

## Abstract

*Candida auris* has emerged as a significant healthcare-associated pathogen due to its multidrug-resistant nature. Ongoing constraints in the discovery and provision of new antifungals create an urgent imperative to design effective remedies to this pressing global blight. Herein, we screened a chemical library and identified aryl-carbohydrazide analogs with potent activity against both *C. auris* and the most prevalent human fungal pathogen, *C. albicans*. SPB00525 [*N*’-(2,6-dichlorophenyl)-5-nitro-furan-2-carbohydrazide] exhibited potent activity against different strains that were resistant to standard antifungals. Using drug-induced haploinsufficient profiling, transcriptomics and metabolomic analysis, we uncovered that Ole1, a Δ(9) fatty acid desaturase, is the likely target of SPB00525. An analog of the latter, HTS06170 [*N*’-(2,6-dichlorophenyl)-4-methyl-1,2,3-thiadiazole-5-carbohydrazide], had a superior antifungal activity against both *C. auris* and *C. albicans*. Both SPB00525 and HTS06170 act as antivirulence agents and inhibited the invasive hyphal growth and biofilm formation of *C. albicans*. SPB00525 and HTS06170 attenuated fungal damage to human enterocytes and ameliorate the survival of *Galleria mellonella* larvae used as systemic candidiasis model. These data suggest that inhibiting fungal Δ(9) fatty acid desaturase activity represents a potential therapeutic approach for treating fungal infection caused by the superbug *C. auris* and the most prevalent human fungal pathogen, *C. albicans*.

## 1 Introduction

*Candida auris* has emerged as a significant healthcare-associated pathogen, posing a serious challenge due to its multidrug-resistant nature. Recent studies emphasized the rapid dissemination of *C. auris* strains across healthcare settings worldwide and its ability to acquire resistance to multiple antifungal agents (Lockhart et al., 2016; Chowdhary et al., 2017; Nelson, 2023). The global impact of *C. auris* outbreaks underscores the critical importance of understanding its resistance mechanisms and developing novel therapeutics. Despite concerted efforts to control its spread, *C. auris* continues to evade traditional infection control measures, posing a significant threat to vulnerable patient populations (Schelenz et al., 2016). This necessitates a multifaceted approach that combines infection prevention strategies with the development of new antifungal agents and treatment modalities.

The management of *C. auris* infections represents a significant clinical challenge due to its resistance to multiple antifungal agents, including azoles, echinocandins, and polyenes (Chowdhary et al., 2017). This multidrug-resistant profile severely limits treatment options and contributes to suboptimal therapeutic outcomes. In clinical practice, the primary therapeutic approach against *C. auris* often involves the utilization of echinocandins such as caspofungin and micafungin. Echinocandins exert their antifungal effects by inhibiting β-1,3-D-glucan synthesis, a crucial component of the fungal cell wall (Szymański et al., 2022). However, despite their efficacy against many *Candida* species, recent studies underscored an increased rate of resistance of *C. auris* isolates to echinocandins (Nelson, 2023). Mutations in *Candida* beta-1,3-glucan synthase genes have been associated with elevated minimum inhibitory concentrations (MICs) to echinocandins, often resulting in treatment failures (Walker et al., 2010; Healey et al., 2020). Furthermore, clinical studies have reported variable responses to echinocandins among different *C. auris* isolates, highlighting the importance of susceptibility testing to guide treatment decisions (Kathuria et al., 2015). In cases where echinocandins are ineffective or contraindicated, alternative antifungal agents such as amphotericin B and azoles may be considered, although their efficacy against *C. auris* strains can be limited, particularly in azole-resistant isolates. Combination therapy, involving the simultaneous use of multiple antifungal agents with distinct mechanisms of action, has been proposed as a potential strategy to enhance treatment efficacy and reduce the risk of resistance emergence (Spivak and Hanson, 2018). Additionally, adjunctive approaches such as the optimization of dosing regimens or the use of novel antifungal formulations, such as lipid-based formulations of amphotericin B, may be explored to improve therapeutic outcomes, particularly in severe or refractory cases of *C. auris* infections (Lamoth, 2023).

Efforts to address the challenge of antifungal resistance in *C. auris* have spurred the development of novel therapeutic agents. These include next-generation azoles, such as isavuconazole and VT-1598, which demonstrate promising activity against azole-resistant strains of *C. auris* (Caballero et al., 2021). Furthermore, the exploration of alternative antifungal classes, such as the development of fungal-specific inhibitors targeting essential cellular processes, holds considerable promise (Jacobs et al., 2022). Compounds targeting fungal cell wall synthesis, mitochondrial function, or stress response mechanisms represent innovative approaches for combatting *C. auris* infections (Lamoth, 2023). High-throughput screening assays have facilitated the identification of novel antifungal compounds with activity against *C. auris*. These screens employ large compound libraries to rapidly assess the inhibitory effects on fungal growth or viability. Recent discoveries have unveiled several lead compounds with potent antifungal activity against *C. auris*, offering new avenues for drug development. Notably, compounds targeting specific vulnerabilities in *C. auris*, such as its unique cell wall composition or metabolic pathways, have emerged as promising candidates (Treviño-Rangel et al., 2022). Additionally, repurposing existing drugs with antifungal properties has garnered attention, leveraging established safety profiles and pharmacokinetic properties to expedite clinical translation.

By screening a chemical library of 678 bioactive molecules, we have identified a series of aryl-carbohydrazides with potent activity against *C. auris* and also *C. albicans*. In particular, SPB00525 [*N*’-(2,6-dichlorophenyl)-5-nitro-furan-2-carbohydrazide] exhibited potent activity against different strains that were resistant to standard antifungal therapeutics. Using drug-induced haploinsufficient profiling, transcriptomics and metabolomic analysis, we uncovered that the Δ(9) fatty acid desaturase Ole1 is the likely target of SPB00525. Moreover, analog HTS06170 [*N*’-(2,6-dichlorophenyl)-4-methyl-1,2,3-thiadiazole-5-carbohydrazide] delivered superior *in vitro* antifungal activity against both *C. auris* and *C. albicans*. Both SPB00525 and HTS06170 inhibited different *C. albicans* virulence traits including the invasive filamentous growth and biofilms, a highly resistant sessile growth associated with the contamination of medical devices and high mortality rates (Vitális et al., 2020). Accordingly, SPB00525 and HTS06170 attenuated fungal damage to human enterocytes and ameliorate survival of *Galleria mellonella* larvae used as a model of systemic candidiasis. These data, suggest that inhibiting Δ(9) fatty acid desaturase activity represents a potential therapeutic approach for treating fungal infection caused by the superbug *C. auris* and the most prevalent human fungal pathogen, *C. albicans*.

## 2 Materials and methods

### 2.1 Fungal strains and growth condition

All fungal strains used in this study are listed in the **Supplementary Table S1**. Ole1 mutant used in this study is a GRACE conditional mutant where one allele was deleted at one locus and the 5’cis-regulatory region of the other locus was replaced by a tetracycline-repressible promoter (Roemer et al., 2003). Conditional repression of Tetracycline-Ole1 promoter was achieved by supplementing growth medium with 100 µg/ml of the antibiotic tetracycline. *C. albicans* Ole1-GFP and Sec61-GFP were constructed by fusing *OLE1* and *SEC61* ORFs excluding the stop codon to GFP using the PCR-based strategy described by Gola et al. (Gola et al., 2003). The fluorescent fusions cassettes were used to transform the *C. albicans* SN148 strain (Noble and Johnson, 2005) using the lithium acetate transformation process. The primer sequences used for the construction of GFP-fusions are listed in the **Supplementary Table S1**. Ole1 and orf19.5258 overexpression constructs were generated by cloning both ORFs in the CIp-Act-cyc plasmid (Blackwell et al., 2003) which was linearized with the *StuI* prior to transformation in the *C. albicans* SN148 strain.

For general propagation, *C. albicans* and *C. auris* strains were routinely maintained at 30°C on YPD (1% Yeast extract, 2% Peptone, 2% Dextrose) or synthetic complete (SC; 0.67% yeast nitrogen base with ammonium sulfate, 2.0% glucose, and 0.079% complete supplement mixture) media supplemented with uridine (50 mg/L). RPMI medium (10.4 g/L RPMI-1640, 3.5% MOPS, 2% D-glucose and 0.3 g/L glutamine from Wisent Inc) was used for the determination of the minimal inhibitory concentration (MIC) of antifungal molecules. The yeast bioactive library included 678 chemical compounds with bioactivity on the budding yeast that were drawn from the Maybridge collection (Wildenhain et al., 2016; Garcia et al., 2018). The SPB00525 chemical analogs A939572 (MolPort-003-983-382), PluriSln1 (MolPort-000-564-458), MK-8245 (MolPort-023-293-543), HTS06170 (MolPort-002-901-421), HTS06154 (MolPort-002-901-407), HTS05029 (MolPort-002-901-093), HTS05668 (MolPort-002-901-311), G514 (MolPort-000-882-466) and new batches of SPB00525 (MolPort-002-923-508) were obtained from MolPort. For growth assay of the conditional shut-off mutant strain of *OLE1* (*ole1*/pTet-*OLE1*), saturated (palmitic and stearic acids, Sigma) and unsaturated (palmitoleic and oleic acids, Sigma) fatty acids, were used at 1.25 mM and 0.5 mM, respectively.

### 2.2 Chemical screen and dose-response assays

The yeast bioactive small molecule library was used to uncover compounds exhibiting antifungal activity against the *C. auris* 381 strain (also called B11220). *C. auris* cells (10^5^ cells/mL) were cultivated in triplicates in a 96-well plate in SC medium, at 30 °C with agitation for 48 hours, using a single concentration of 100 µM for each compound. The optical density at 600 nm (OD_600_) was measured each 10 min using a Sunrise plate-reader (Tecan). The percentage growth of each species for a given compound was calculated relative to the average growth observed in the DMSO control wells on each plate. Compounds displaying 50% growth inhibition were selected and underwent another round of screening under identical conditions to confirm bioactivity.

Antifungal activity of small molecules was evaluated through dose-response assays conducted in 96-well flat-bottom microtiter plates (Thermo Fisher Scientific). Fungal strains were grown overnight in SC medium at 30°C in a shaking incubator at 220 rpm. Cells were then resuspended in fresh SC at an OD_600_ of 0.05. A total volume of 99 μl fungal cells was added to each 96-well plate in addition to 1 μl of the corresponding stock solution to get the following concentrations of each bioactive molecule: 3, 6, 9, 12, 15, 30 and 60 µg/ml. Plates were incubated in a plate reader (Sunrise, Tecan) at 30°C with agitation and OD_600_ readings were taken every 10 min over 48h. Each experiment was performed in triplicate. For each concentration, growth inhibition was calculated as a percentage of OD_600_ measurements of treated-fungal cells to that of the control experiment (DMSO-treated cells).

### 2.3 Minimal inhibitory and minimal fungicidal concentrations determination

The minimal inhibitory concentrations (MIC) were determined following Clinical and Laboratory Standards Institute (CLSI) recommendations using RPMI liquid medium. 96-well plates were incubated at 30°C with shaking and OD_600_ readings were taken after 24 h. The MIC was determined as the first well with growth reduction of >50% based on OD_600_ values in the presence of the tested molecules as compared to untreated control cells.

Minimal fungicidal concentrations (MFC) was determined as previously described by Chaillot et al. (Chaillot et al., 2017b). Cells of overnight cultures of *C. auris* 381 and *C. albicans* SC5314 strains were counted using a hemocytometer and diluted to a concentration of 10^6^ cells/ml. Cells were grown in RPMI at 30° C under shaking in the presence of different concentration of SPB00525 (3-15 µg/ml). Samples from these cultures were periodically withdrawn and plated onto YPD plates at 24h after treatment. Different dilutions were made to achieve a good count of colony-forming units (CFU). Cultures displaying low viability were plated without further dilution. The kill curves were constructed by calculating the average of the CFU per milliliter surviving at each concentration of the tested molecules.

### 2.4 SPB00525-induced haploinsufficiency profiling

The double-barcoded heterozygote *C. albicans* (DBC) mutants were obtained from the NRC’s Royalmount Avenue Research Facility (Montreal, Canada). A 384-well pin tool was used to transfer DBC mutant cells into OmniTrays single-well plate containing YPD-agar, and colonies were grown for 48 hr at 30°C. Missing or slow growing colonies were grown separately by streaking 100 µl of the initial liquid cultures. Strains were then scrapped from the plates and pooled togethers in YPD + 15% glycerol and stored in aliquots at -80°C. Aliquots were diluted to OD_600_ = 0.062 and exposed or not to 6 µg/ml SPB00525 in either SC or YPD and grown at 30°C under agitation for 15 hours. Of note, 6 µg/ml SPB00525 led to a similar growth inhibition rate (20%) in both YPD and SC media. 1 mL of the sub-cultured pool was distributed into duplicate culture tubes, each containing 1 mL of YPD or SC medium supplemented with either 6 µg/ml SPB00525 or DMSO solvent. These cultures were incubated at 30°C under shaking conditions for 24 hours. A total of two biological replicates were considered. Cells were harvested by centrifugation and genomic DNA was extracted using YeaStar kit (Zymo Research). The Up-tag DNA-barcodes were amplified using 25 ng genomic DNA with DBC-F1 and DBC-R1 primers that recognize the common region of up-tag barcode and contain the multiplexing tag (DBC-F1) and sequences required for hybridization to the Illumina flow cell (**Supplementary Table S1**). PCR amplification products were purified form an agarose gel using the QIAquick Gel Extraction kit (Qiagen) and quantified by QuantiFluor dsDNA System (Promega). Equal quantity of DNA from the SPB00525-treated and non-treated pools were combined prior to NGS sequencing using Illumina MiSeq platform and DBC1 and DBC2 sequencing primers listed in the **Supplementary Table S1**. Barcode-sequencing data was processed as described previously (Chaillot et al., 2017a). Unique uptag DNA barcodes were extracted and counted using SeqKit (Shen et al., 2016). The fitness scores reflect the differential abundance of each uptag barcode/mutant strain in the SPB00525-treated condition relative to the DMSO control. For each mutant, a fitness score that reflects the differential abundance in the SPB00525-treated condition relative to the DMSO control was calculated for each growth medium. Mutants in **Figures 2C, D** were identified using Welch’s t test with a false-discovery rate of 5% and a 2.5-fold (Log_2_) enrichment cutoff. Barcode-sequencing data have been submitted to the GEO database under accession number GSE267057.

### 2.5 Expression analysis by RNA-seq

Overnight cultures of *C. albicans* SC5314 and *C. auris* 381 strains were diluted to an OD_600_ of 0.1 in 50 ml of fresh SC medium and grown at 30°C under agitation (200 rpm) to early logarithmic phase (OD_600 =_ 0.6). Cultures were then either left untreated or exposed to 6 µg/ml SPB00525 and incubated at 30°C for 15- and 60-min. Cells were harvested by centrifugation and were flash-frozen and stored at -80°C. For each condition, a total of two biological replicates were considered for RNA-seq analysis. Total RNA was extracted using an RNAeasy purification kit (Qiagen) and glass bead lysis in a Biospec Mini 24 bead-beater as previously described (Tebbji et al., 2014). RNA integrity was assessed using Agilent 4200 Tape Station System prior to cDNA library preparation. The NEBNext Ultra II RNA Library Prep Kit for Illumina was used to construct the RNA-seq library following the manufacturer’s instructions. A paired-end sequencing of cDNAs was performed using an Illumina NovaSeq 6000 sequencing system. Sequencing reads were filtered with fastp version 0.23.2 (Chen et al., 2018) and mapped to the reference genome sequence with STAR version 2.7.9a (Dobin et al., 2013). The number of reads mapping on each of the genes was calculated by featurecount version 2.0.1 (Liao et al., 2014). Differential expression of genes was calculated with DESeq2 version 1.40.1 (Love et al., 2014). Differentially expressed transcripts in the **Supplementary Table S3** were identified using Welch’s t-test with a false-discovery rate (FDR) of 1% and 1- Log_2_ fold enrichment cut-off. Gene ontology (GO) analysis was performed using GO Term Finder of the Candida Genome Database (Binkley et al., 2014). The GSEA (Gene Set Enrichment Analysis) pre-ranked tool (http://www.broadinstitute.org/gsea/) was used to determine statistical significance of correlations between the *C. albicans* Mn-sensitive transcriptomes with GO biological process terms and different omics datasets as described in (Sellam et al., 2012; Burgain et al., 2019). All RNA-seq raw data are available in **Supplementary Table S3**. RNA-seq data have been submitted to the GEO database under accession number GSE267057.

### 2.6 Fatty acid quantification

Fatty acids were quantified by gas chromatography-Mass spectrometry as previously described (Montreal Heart Institute metabolomics facility, Montreal, Canada) (Turcot et al., 2015). Briefly, overnight cultures of *C. albicans* SC5314 strains were diluted to an OD_600_ of 0.1 in 40 ml of fresh SC medium and incubated at 30°C under agitation (200 rpm) until reaching early logarithmic phase (OD_600 =_ 0.3). At this point, cultures were either left untreated or exposed to 9 or 30 µg/ml SPB00525 and further incubated at 30°C for 2.5 hours. Subsequently, cells were washed twice with PBS and then frozen. Metabolites extraction was performed for each condition, with a total of five biological replicates. Fatty acid esters were injected into a GC/MS (Agilent 6890 gas chromatograph coupled to a 5975N mass selective detector) that was operated in a chemical ionisation mode with ammonia as the reagent gas. Fatty acids were then separated in a Varian CP7420 FAME polar capillary column and analyzed as their [M+NH4]+ ions. Fatty acid ions were identified according to their retention time and their concentrations were calculated using internal/external labelled standards. The concentration of each individual FA (C16:0, C18:0, C16:1n7, C18:1n9) was expressed as the mean percentage of total FA concentration in five biological replicates.

### 2.7 Evaluation of antivirulence activity

For the biofilm assay, overnight cultures of *C. albicans* SC5314 grown in SC medium were rinsed twice with PBS and then suspended in RPMI 1640 supplemented with L-glutamine to have an OD_600_ of 0.1. Cell suspension (100 µl) was added to each well of 96-well polystyrene plates and incubated at 37°C for 2 hours to initiate biofilm formation. After three washes to remove non-adherent cells, fresh RPMI medium alone or containing specified concentrations of drugs (SPB0500 or HTS06170) was added to the wells. The plates were further incubated at 37°C for 24 hours with shaking at 75 rpm. Following drug treatment, non-adherent cells were removed by three gentle washes with 1x PBS. Biofilm biomass was determined using the crystal violet assay as described by Regan et al. (Regan et al., 2017). *C. albicans* biofilms were air-dried for 45 minutes, stained with 0.4% aqueous crystal violet solution for 45 minutes at room temperature, washed twice with sterile water, and then de-stained with 95% ethanol for 45 minutes at room temperature. The de-stained solution was diluted 1:10 in 95% ethanol, and the absorbance was measured at OD_595_.

Filamentation was assessed by exposing *C. albicans* SC5314 yeast cells to various filament-inducing conditions. The cells were initially cultured overnight in SC medium until saturation was achieved. Subsequently, the cells were diluted to a concentration of 1x10^5^ cells/ml in different growth media: SC medium alone, SC medium supplemented with 10% fetal bovine serum (FBS) and Spider, lee’s or RPMI media. A volume of 500 µl of cell suspension was dispensed into wells of a 24-well plate, along with the specified concentrations of SPB0500 or HTS06170. The plates were then incubated at 37°C. Following a 6-hour incubation period, images of the cells were captured using the high-content microscope Cytation 5 and the length of the filament was measured using ImageJ software (Schneider et al., 2012).

### 2.8 Galleria virulence assay

*Galleria mellonella* Larvae (Elevages Lisard, Canada) were used at their instar developmental stage. *C. albicans* SC5314 strains from overnight cultures were subjected to double washing and then diluted in 20 µl of PBS to get 5 × 10^5^ cells for injection. Larvae weighing 180 ± 10 mg were injected at the third pair of prothoracic legs. The injection was administered on the right side for *C. albicans* and on the left side for either 15 µg/ml of SPB0500 or HTS06170. The infected larvae were subsequently kept at 37°C. Two sets of replicates, each comprising 40 larvae, were used with survival rates assessed each 12 hours over a 4-day period. Control experiments encompassing DMSO injection, or injection of SPB0500 or HTS06170 alone without *C. albicans* were conducted using 30 larvae. Death was determined by the absence of response to touch and the inability to right themselves. Kaplan-Meier survival curves were constructed and subjected to comparison utilizing the log-rank test (GraphPad Prism 5).

### 2.9 HT-29 damage assay

Epithelial cell line HT29 (ATCC; HTB-38) was acquired from the American Type Culture Collection (ATCC). Cells were cultured in McCoy’s Medium (Wisent # 317-010 CL) supplemented with10% heat-inactivated fetal bovine serum (FBS, Sigma). Cultures were maintained at 37°C in a 5% CO_2_ environment. Experiments were conducted within 12 serial passages from thawing. The damage inflicted on the human colon epithelial cell line HT-29 was evaluated using a lactate dehydrogenase (LDH) cytotoxicity detection kitPLUS (Roche), which quantifies the release of the LDH enzyme into the growth medium.

HT-29 cells were cultured in 96-well plates as monolayers in McCoy’s medium supplemented with 10% FBS) at a density of 2 × 10^4^ cells per well. The cultures were then incubated overnight at 37°C with 5% CO_2_. Subsequently, the cells were infected with *C. albicans* SC5314 or *C. auris* 381 cells that had been precultured in SC medium, at a multiplicity of infection (MOI) of 1:5 (cell:yeast) for 24 hours at 37°C with 5% CO_2_. After the incubation period, 100 µl of supernatant was collected from each experimental well, and LDH activity was assessed by measuring absorbance at 490 nm in accordance with the manufacturer’s instructions. Cytotoxicity was determined using the following formula: % cytotoxicity = [experimental value − low control (untreated cells)]/[high control (1% Triton X-100) − low control] × 100.

## 3 Results

### 3.1 Small molecule screen identifies SPB00525 as potent antifungal agent against *C. auris*

**Figure 1:**
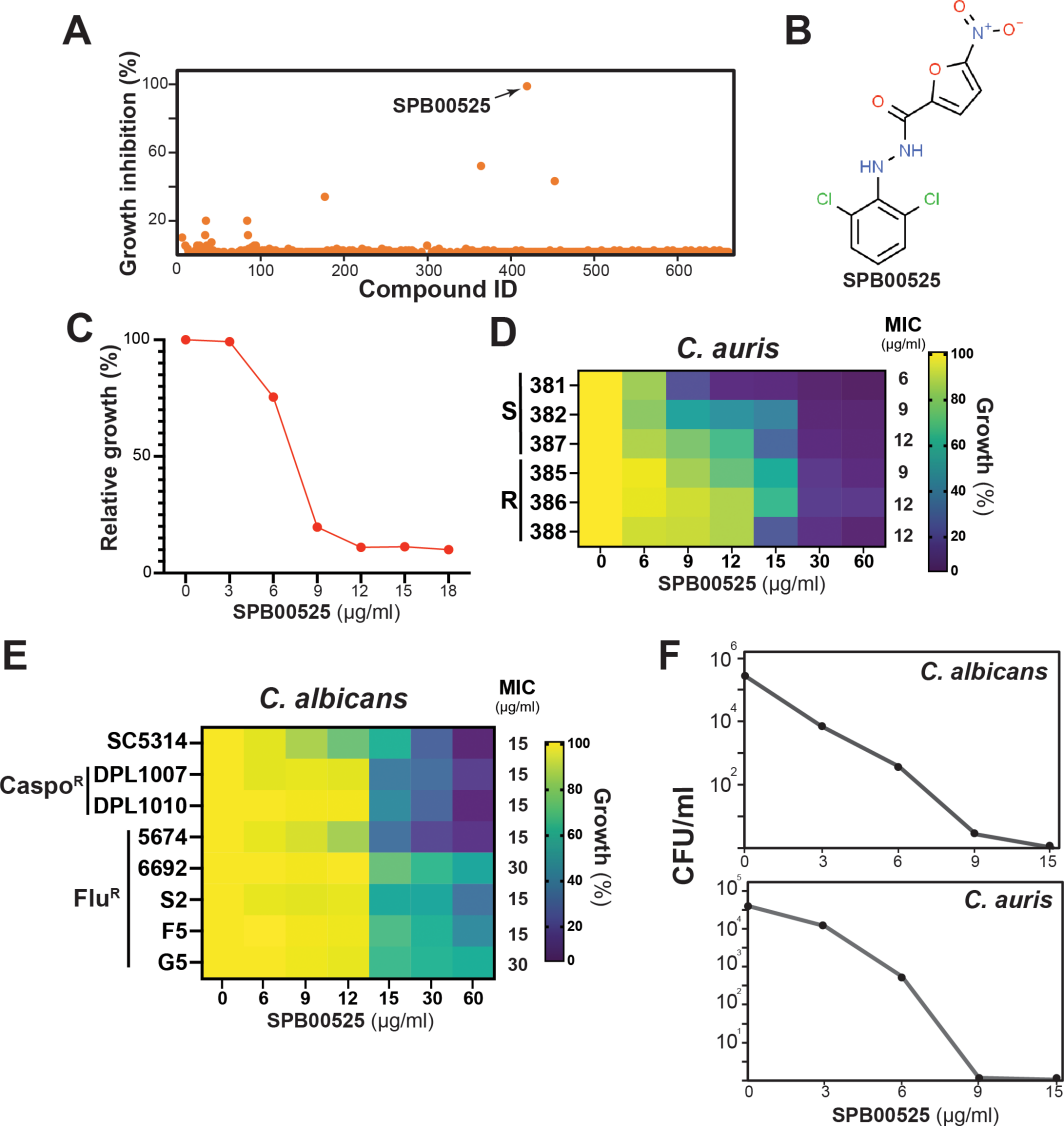
Library screen data, hit validation, and antifungal activity of SPB00525. **(A)** Screen of the yeast bioactive library against molecules with antifungal activity against *C. auris*. Relative inhibition of each compound tested at 100 µM was determined on *C. auris* 381 isolate grown at 30°C in SC medium for 24 h. **(B)** Chemical structure of the aryl-carbohydrazide hit SPB00525. **(C)** Dose-response assay of SPB00525 on *C. auris*. The *C. auris* 381 strain was grown in SC medium with different concentration of SPB00525 and OD reading was taken after 24h of incubation. Results represent growth inhibition (%) relative to the DMSO control. **(D)** Heat map representing the dose-response assay of SPB00525 on different azole resistant (R) and sensitive (S) clinical isolates of *C. auris*. The minimal inhibitory concentrations (MIC) were determined following Clinical and Laboratory Standards Institute (CLSI) recommendations using RPMI medium. **(E)** Heat map of the dose-response assay of SPB00525 on sensitive and, azole- (Flu^R^) and caspofungin-resistant (Caspo^R^) clinical isolates of *C. albicans*. **(F)** Time-kill curve showing the fungicidal activity of SPB00525 against both *C. auris* and *C. albicans*. *C. auris* 381 and *C. albicans* SC5314 strains were exposed to different concentrations (3-15 µg/ml) at 24h. CFUs were calculated as described in the method section.

To identify novel compounds with antifungal activity against *C. auris*, we screened a library of 678 yeast bioactive small molecules. This library contains molecules that were selected from a 50,000 compound Maybridge library based on bioactivity in *S. cerevisiae*, as well as their balance of hydrophilic and lipophilic properties (Ishizaki et al., 2010; Wong et al., 2013; Garcia et al., 2018). *C. auris* 381 isolate cells were grown in 96-well plates in triplicate under a yeast-promoting condition (SC medium) for 48 hours with a concentration of 100 µM screening compound. Aryl-carbohydrazide SPB00525 emerged as a hit that completely inhibits the growth of *C. auris* (**Figures 1A, B**). The antifungal activity of SPB00525 was also recapitulated using new batches from alternative suppliers. SPB00525 reduced the growth of *C. auris* in a dose-dependent manner with a MIC of 6 µg/ml (18 µM) (**Figure 1C**). SPB00525 was also active against different sensitive and fluconazole-resistant *C. auris* clinical isolates with MICs ranging from 6-12 µg/ml (**Figure 1D**). Moreover, SPB00525 retained moderate activity against different clinical isolates of the most prevalent human fungal pathogen, *C. albicans*, regardless their antifungal sensitivity profiles, and with MIC of 15-30 µg/ml (**Figure 1E**). SPB00525 showed fungicidal activity toward *C. auris* and *C. albicans* with minimal fungicidal concentrations (MFC) of 9 µg/ml and 15 µg/ml, respectively (**Figure 1F**). Overall, our chemical screen identified a novel antifungal compound with antifungal activity against both sensitive and resistant isolates of *C. auris* and *C. albicans*.

### 3.2 Chemical genetic profiling uncovers the Δ(9) fatty acid desaturase Ole1 as a potential target of SPB00525

**Figure 2:**
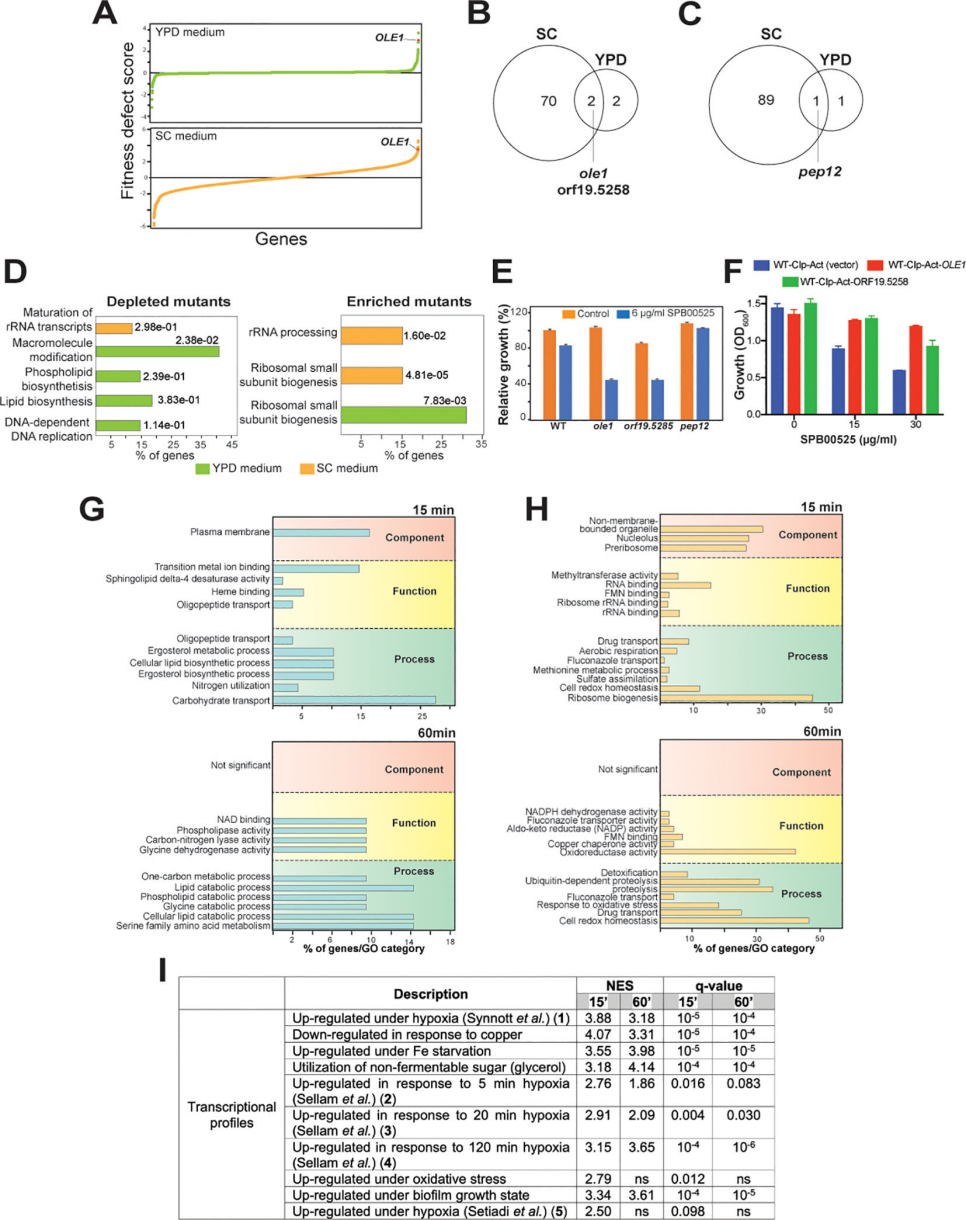
Haploinsufficient profiling and RNA-seq assays uncover the Δ(9) fatty acid desaturase Ole1 as a potential target of SPB00525. **(A)** GO biological process term enrichment of *C. albicans* heterozygous mutants depleted or enriched upon exposure to SPB00525 in both SC and YPD media. **(B)** Graphical representation of results from the SPB00525-induced haploinsufficiency profiling. Mutants were plotted according to their fitness defect score from lowest to highest. The fitness scores reflect the differential abundance of each mutant strain in the SPB00525-treated condition relative to the DMSO control. **(C, D)** Comparison of HIP profiles in both SC and YPD growth media. Venn diagrams indicate shared depleted **(C)** and enriched **(D)** mutants in the presence of SPB00525 in SC and YPD media. SPB00525- enriched and depleted mutants were identified using a fitness score cutoff of 2.5 (Log_2_) and a false discovery rate of 5%. **(E)** Validation of the SPB00525-induced haploinsufficiency assay. WT (CAI4) and heterozygous mutants (*ole1*, *orf19.5285* and *pep12*) cells were grown in SC medium and exposed or not to 6 µg/ml SPB00525. Results represent growth inhibition (%) relative to the DMSO control. **(F)** Increased dosage of *OLE1* and orf19.5285 led to decreased SPB00525 sensitivity of *C. albicans*. WT (WT-Cip-Act) and strains overexpressing both *OLE1* and orf19.5285 were exposed to 15 and 30 µg/ml SPB00525 and OD_600_ reading were acquired at 24h. **(G, H)** Transcriptomic analysis of *C. albicans* response to SPB00525. GO enrichment of upregulated **(G)** and downregulated **(H)** transcripts of *C. albicans* cells exposed to 6 µg/ml SPB00525 for 15 and 60 min. **(I)** Gene set enrichment analysis (GSEA) of the *C. albicans* SPB00525-modulated transcriptome at 15- and 60-min. NES (normalized enrichment score) and nominal *q*-value obtained from the GSEA are shown for each correlation. The complete GSEA correlations are listed in **Supplementary Table S4**. False-Discovery Rate (*q*-value) of 1%.

To uncover the potential target of SPB00525, we used the drug-induced haploinsufficient profiling (HIP) assay using the *C. albicans* barcoded-heterozygous deletion collection (Xu et al., 2007). The principal of this assay is based on the fact that decreased dosage of a drug target gene in a heterozygous mutant can result in increased drug sensitivity (Giaever et al., 1999; Hoon et al., 2008). Strains of the DNA-barcoded *C. albicans* heterozygous mutants were pooled and grown in the absence or the presence of 6 µg/ml SPB00525. To rule out media composition bias on the sensitivity of pooled mutants to SPB00525 (Chang et al., 2010), the HIP assays were performed both in the complex YPD and the synthetic SC media. The fitness scores reflecting the differential abundance of each mutant in the SPB00525-treated condition relative to the DMSO control in each growth medium was calculated. Using a fitness score cutoff of 2.5 (Log_2_) and a false discovery rate of 5%, a total of 72 and 3 strains were depleted after the exposure to SPB00525 in both SC and YPD media, respectively. GO-term analysis of genes whose mutation caused significant growth defects toward SPB00525 revealed a difference in the overrepresented GO terms between the two-growth media with processes related to lipid metabolism being enriched in YPD and rRNA maturation processes in SC (**Figure 2A**; **Supplementary Table S2**). The set of genes whose deletion conferred enhanced fitness regardless growth medium (90 and 2 in SC and YPD growth media, respectively) was associated with ribosome biogenesis and rRNA processing. Despite this disparity, two mutants were depleted in both growth conditions including the mutant of the Δ(9) fatty acid desaturase (*ole1/OLE1*) and a mutant of the uncharacterized gene orf19.5258 (**Figures 2B, C**). The hypersensitivity of the two heterozygous deletion mutants was individually confirmed suggesting that either Ole1 and/or orf19.5258 might be a target of SPB00525 (**Figure 2E**). Accordingly, overexpression of either *OLE1* or orf19.5258 attenuate the antifungal activity of SPB00525 (**Figure 2F**). The only mutation that buffered the growth of *C. albicans* to SPB00525 in both SC and YPD medium was a heterozygous mutation of *PEP12*, a gene required for vesicular transport between the Golgi apparatus and the vacuole (**Figures 2D, E**).

Transcriptional profiling using RNA-seq was used to capture the cellular response of both *C. albicans* and *C. auris* to SPB00525 at two different time-points (15 and 60 min). Exposure of both yeasts to SPB00525 resulted in differential modulation of different metabolic processes reflecting a reprogramming of fungal metabolism (**Figures 2G, H**; **Supplementary Figure S1**; **Supplementary Table S3**). Overall, in *C. albicans*, transcripts associated with carbohydrate transport, lipid metabolisms (ergosterol and phospholipid) and utilization of nitrogen sources were downregulated while genes of drug transport and oxidative stress were induced (**Figures 2G, H**). A similar trend was perceived in *C. auris* with a differential modulation of transcripts related to ATP generation, beta-oxidation, glycolysis, lysine synthesis and glycogen and trehalose biosynthesis (**Supplementary Figure S1**). Intriguingly, GSEA analysis of the SPB00525-modulated transcript revealed similarities with transcriptional signatures of *C. albicans* cells exposed to hypoxia in different independent investigations (Setiadi et al., 2006; Synnott et al., 2011; Sellam et al., 2014) (**Figure 2I**; **Supplementary Figure S3**). Given the requirement of oxygen for fatty acid desaturase catalysis (Los and Murata, 1998), this finding further supports that SPB00525 act as Ole1 inhibitor as hypoxia led to mono- and poly-unsaturated fatty acids depletion in *C. albicans* (Burgain et al., 2020).

At 60 min exposure SPB0052, both *C. albicans* and *C. auris* cells upregulate genes of proteolysis, protein refolding and redox homeostasis which is reminiscent of the unfolded protein response (UPR) and might reflect ER stress (**Figure 2H**; **Supplementary Figure S1**; **Supplementary Table S3**). Fatty acid desaturases such as Ole1 are endoplasmic reticulum-bound enzyme that catalyzes the desaturation of saturated fatty acyl-CoA (Miyazaki and Ntambi, 2003). To rule out that inhibition of Ole1 is a consequence of structural perturbation of ER by SPB0052 which in turn might lead to inappropriate functioning of this enzyme, we assessed ER integrity using a *C. albicans* strain expressing the Sec61-GFP (Manford et al., 2012; Chaillot et al., 2015). Integrity of either nuclear or cortical ER was preserved even at sublethal concentration of SPB0052 supporting a direct inhibition of Ole1 by this small molecule rather than an indirect consequence of ER breakdown (**Supplementary Figure S2**).

### 3.3 Inhibition of Ole1 desaturase by SPB00525

**Figure 3:**
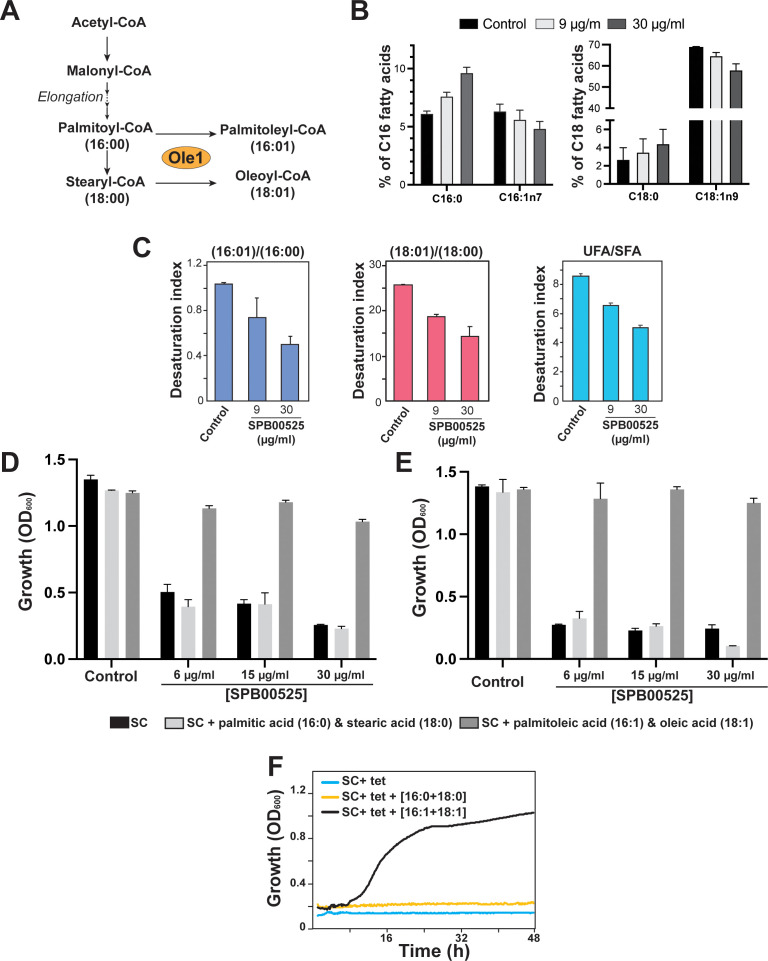
Inhibition of Ole1 desaturase activity by SPB00525. **(A)** Desaturation of fatty acyl CoAs by fungal Δ(9) fatty acid desaturase. Ole1 converts CoA-linked saturated (palmitoyl-CoA and stearyl-CoA) to monounsaturated fatty acids of 16 (palmitoleic acid) or 18 (oleic acid) carbons in length. **(B, C)** SPB00525 inhibits fatty acid desaturation. SPB00525 reduced both C16 and C18 desaturation **(B)**. **(C)** Fatty acid desaturation index (unsaturated/saturated ratio) of C16, C18 and total fatty acid of *C. albicans* SC5314 strain exposed to 9 and 30 µg/ml SPB00525 for 2.5 hours. Data are average of five experiments, and error bars are SD. UFA: Unsaturated fatty acids; SFA: Saturated fatty acids. **(D, E)** Supplementation of exogenous unsaturated fatty acids alleviates growth inhibition by SPB00525. *C. albicans* SC5314 **(D)** and *C. auris* 381 **(E)** cells were exposed to SPB00525 in SC medium supplemented with either saturated (palmitic + stearic acids) or unsaturated (palmitoleic and oleic acids) fatty acid mixture. **(F)** *OLE1* essentiality was reverted by supplementation of an equimolar mixture of palmitoleic and oleic acids. The conditional shut-off mutant strain of *OLE1* (*ole1*/pTet-*OLE1*) was grown in SC medium with tetracycline (SC+tet) supplemented with saturated or unsaturated fatty acids. Cells were grown at 30°C, and OD_600_ readings were taken every 15 min for 2 days.

Fungal Δ(9) fatty acid desaturases convert palmitoyl- (16:0) and stearoyl- (18:0) CoA to palmitoleic (16:1) and oleic (18:1) fatty acids, respectively (Martin et al., 2007) (**Figure 3A**). To support Ole1 as the target of SPB00525, we assessed its effect on Ole1 desaturase activity by measuring the abundance of both saturated and unsaturated fatty acids using gas chromatography-mass spectrometry (GC-MS). SPB00525 treatment decreased significantly the monounsaturated fatty acids palmitoleate and oleate and increased their saturated counterparts, palmitate, and stearate (**Figure 3B**). Desaturation index (unsaturated to saturated fatty acid ratio) for C16, C18 and for all detected fatty acids were significantly reduced in a dose-dependent manner (**Figure 3C**). At 30 µg/ml, SPB00525 reduced the desaturation index of C16 and C18 fatty acids by 51% and 44%, respectively (**Figure 3C**). These data provide a further support for Ole1 as a direct target of SPB00525. In accordance with this finding, our data showed that growth inhibition of *C. albicans* and *C. auris* by SPB00525 was alleviated by supplementing the growth medium with an equimolar mixture of palmitoleic and oleic acids (**Figures 3D, E**). Similarly, *OLE1* essentiality was also reverted by palmitoleic and oleic acids supplementation (**Figure 3F**). Meanwhile, adding the saturated counterparts of the aforementioned fatty acids has no impact on the inhibitory effect of SPB00525 on *C. albicans*. Taken together, these experiments indicate that SPB00525 inhibits Ole1-mediated fatty acid desaturation.

### 3.4 Aryl-carbohydrazide analogs exhibit antifungal activity

**Figure 4:**
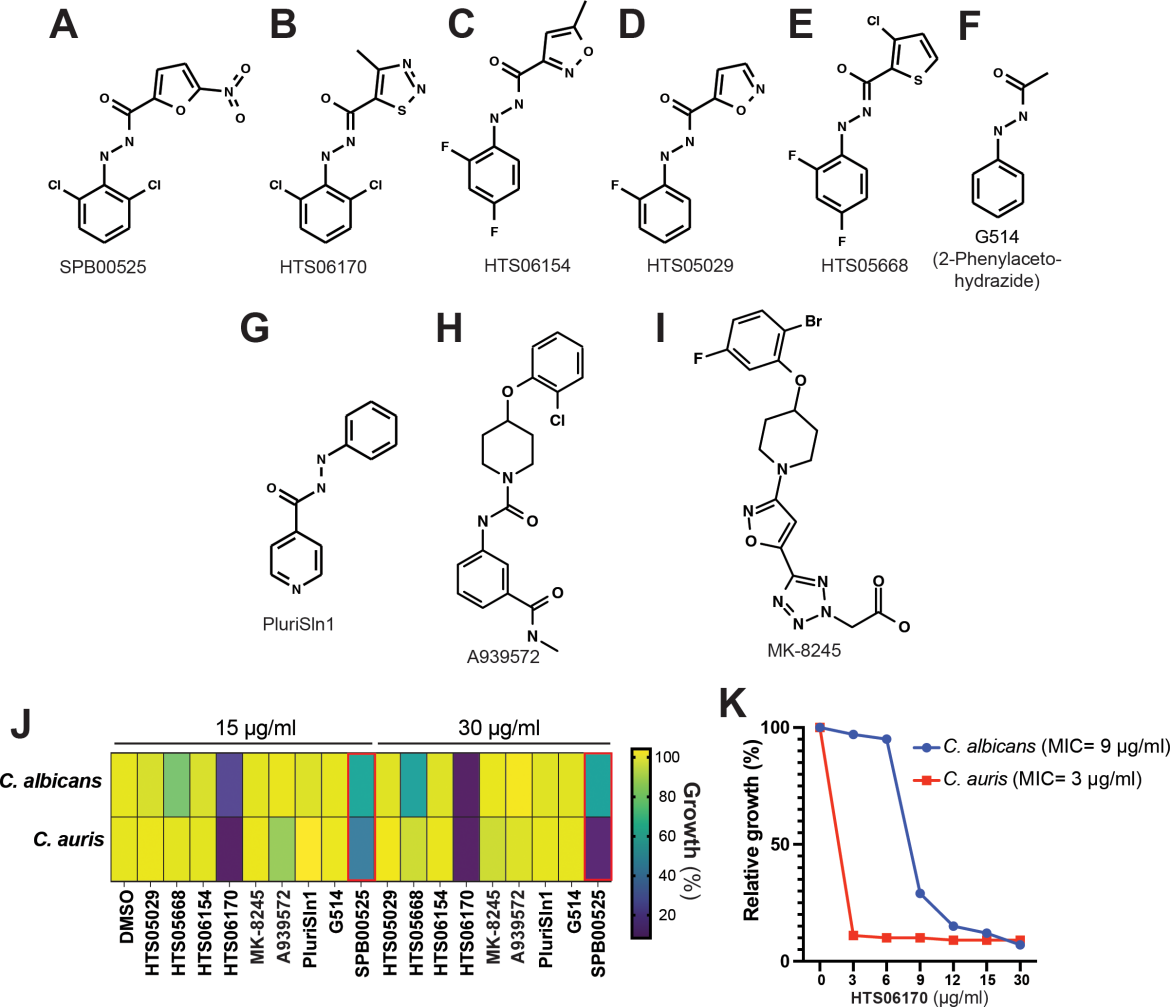
HTS06170 chemical analog of SPB00525 exhibit potent antifungal activity *in vitro*. **(A-F)** Chemical structure of commercially available analogs of SPB00525. **(G-I)** Chemical structure of known human stearoyl-CoA desaturase 1 inhibitors. **(J)** Antifungal activity of SPB00525 analogs tested at 15 and 30 µg/ml. *C. albicans* SC5314 and *C. auris* 381 strains were grown in SC medium and exposed to the corresponding compound for 24h at 30°C. Data reported as percentage growth relative to the untreated condition (DMSO). **(K)** Dose-response assay of HTS06170 on *C. albicans* and *C. auris*. The *C. auris* 381 and the *C. albicans* SC5314 strains were grown in SC medium with different concentration of HTS06170 and OD reading was taken after 24h of incubation at 30°C. Results represent growth inhibition (%) relative to the DMSO control. MICs of HTS06170 are indicated in parentheses for each fungal strain.

Five commercial analogs of SPB00525 and three known human stearoyl-CoA desaturase 1 (Scd1) inhibitors were purchased and tested for antifungal activity against both *C. albicans* and *C. auris* (**Figures 4A–I**). Potency was lost relative to SPB00525 in all cases except with HTS06170 (**Figure 4J**), which displayed improved antifungal activity on both *C. auris* and *C. albicans* with MICs of 3 and 9 µg/ml, respectively (**Figure 1K**). The growth of *C. albicans* and *C. auris* was marginally affected by the three Scd1 inhibitors A939572, PluriSln1 and MK-8245 (**Figures 4G–I**). Although stearoyl-CoA desaturases are similar to their metazoan counterparts [~32% (DeJarnette et al., 2021)], these results suggest that divergence is sufficient to design specific inhibitors for fungal and mammalian desaturases. Notably, although PluriSln1 (Ben-David et al., 2013) exhibits structural similarity to both SPB00525 and HTS06170, no detectable antifungal activity was observed.

### 3.5 SPB00525 and HTS06170 both exhibit *in vivo* antifungal activity and negligible cytotoxicity in human cell lines

**Figure 5:**
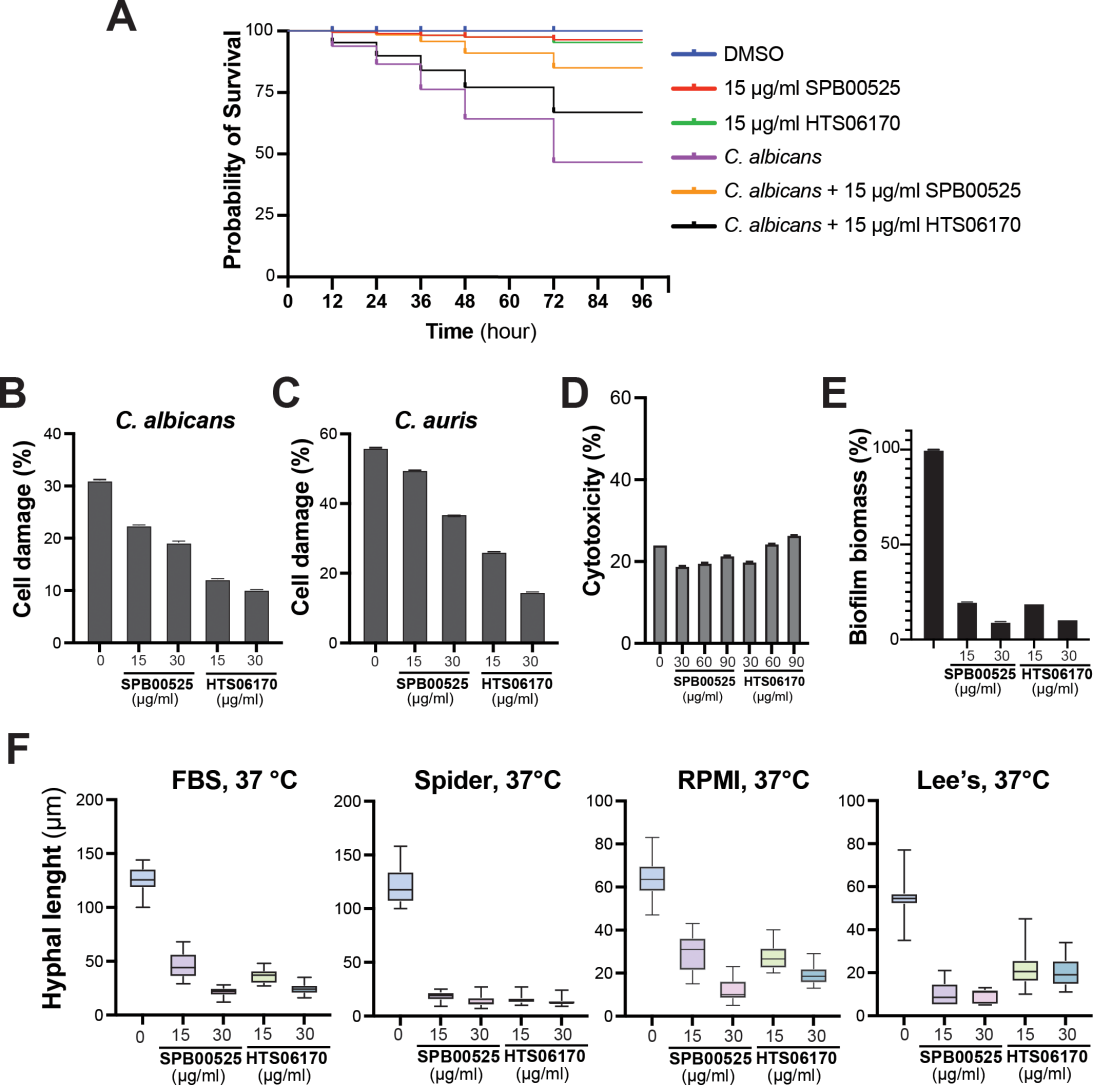
SPB00525 and HTS06170 exhibit *in vivo* activity against *C. albicans* and *C. auris*. **(A)** SPB00525 and HTS06170 attenuate candidiasis in *Galleria mellonella*. Larvae were first injected with *C. albicans* SC5314 strain followed by a second injection of either DMSO, 15 µg/ml of SPB00525 or HTS06170 and survival was monitored at the indicated time for a period of 4 days. **(B, C)** SPB00525 and HTS06170 attenuate damage of enterocytes cells caused by *C. albicans* **(B)** and *C. auris* **(C)**. Damage of the human epithelial intestinal cells HT-29 infected by *C. albicans* SC5314 or *C. auris* 381 strain was assessed using LDH release assay. Cell damage was calculated as percentage of LDH activity of each treatment to that of the control experiment (HT-29 damaged by 1% Triton X-100). Results are represented as the mean of three independent replicates. **(D)** Evaluation of SPB00525 and HTS06170 cytotoxicity using the LDH release assay. HT-29 cells were exposed to 2x, 4x and 6x MIC*_C. albicans_* or 5x, 10x and 15x MIC*_C. auris_* of both SPB00525 and HTS06170 for 24h. **(E)** SPB00525 and HTS06170 inhibit biofilm formation. *C. albicans* SC5314 cell suspension was seeded into 96-well polystyrene plates and incubated at 37°C for 2 hours to initiate biofilm formation. Biofilms were then exposed or not to either SPB00525 or HTS06170 and incubated for 24 hours prior to biomass assessment using the crystal violet assay. Results are represented as the mean of six independent replicates. **(F)** SPB00525 and HTS06170 inhibit filamentation of *C. albicans* in response to different stimuli. *C. albicans* SC5315 yeast cells was grown under hyphae-promoting conditions (FBS and Spider, RPMI or Lee’s media) at 37°C for 6 hours in the absence or the presence of aryl-carbohydrazides. Filament lengths were measured for at least 100 cells using ImageJ software.

The *in vivo* antifungal activity of SPB00525 and HTS06170 was assessed using the *Galleria mellonella* model of systemic fungal infection. *G. mellonella* larvae were infected with the *C. albicans* SC5314 strain and treated with either 15 µg/ml SPB00525 or HTS06170. Administration of only SPB00525 or HTS06170 to larvae resulted in a similar pattern of viability as the *C. albicans*-free DMSO control, with only 1% mortality at 72 hours. While *C. albicans* infection caused 54% mortality at 72 hours, injection of SPB00525 and HTS06170 to infected larvae significantly reduced the mortality rate to 11% and 33%, respectively (**Figure 5A**). The protective effect of SPB00525 and HTS06170 was further tested on human colon epithelial HT-29 cells infected with either *C. albicans* or *C. auris* using the LDH release assay. Our data demonstrated that both SPB00525 and HTS06170 significantly decreased the damage caused by the two yeasts to HT-29 enterocytes (**Figures 5B, C**). Notably, HTS06170 exhibited greater protective activity compared to SPB00525 at the two tested concentrations against both *C. albicans* and *C. auris* (**Figures 5B, C**). These data confirmed that the *in vitro* antifungal activity of SPB00525 and HTS06170 translated to an *in vivo* protective effect on host cells. Furthermore, both SPB00525 and HTS06170 exhibited negligible toxicity on HT-29 cells when tested at 3x, 6x and 10x MIC*_C. albicans_* or 5x, 10x and 15x MIC*_C. auris_* (**Figure 5D**).

We also tested the effect of SPB00525 and HTS06170 on the inhibition of *C. albicans* virulence traits including biofilm formation and differentiation of invasive hyphae. Both SPB00525 and HTS06170 impaired the ability of *C. albicans* to form biofilm leading to a biomass reduction of 80% and 91% at 15 and 30 µg/ml, respectively (**Figure 5E**). Both compounds inhibited hyphae formation triggered by different filamentation-promoting cues (FBS and Spider, RPMI or Lee’s media) (**Figure 5F**). Overall, our data demonstrate that SPB00525 and HTS06170 display antivirulence activity against *C. albicans*, which may explain their antifungal protective effect on host cells.

## 4 Discussion

*C. auris* is an emerging pathogen listed in the WHO critical priority group due to its serious global health threat. Since 2009, *C. auris* has been causing systemic infections in patients with pre-existing health conditions, with a negative prognosis due in part to the broad resistance to standard antifungals. Hence, it is crucial to identify new drug targets for innovative antifungal therapies against this superbug. Herein, we identified two analogs bearing a common aryl-carbohydrazide scaffold that were highly potent against *C. auris* and also *C. albicans*. SPB00525 was effective against echinocandin-resistant isolates of *C. albicans*, as well as azole-resistant strains of both *C. auris* and *C. albicans*. SPB00525 could thus be potentially used to address refractory fungal infections caused by resistant strains. Furthermore, common pleiotropic resistance mechanisms (overexpression of efflux pumps and Pdr16), such as those of resistant strains tested here, likely do not alter fungal susceptibility to SPB00525.

Interestingly, both SPB00525 and HTS06170 were active against *C. albicans* biofilms in a manner that is similar to their activity against planktonic cells. Given that biofilm growth is resistant to conventional antifungals, SPB00525 and HTS06170 may therefore represent promising alternatives for antibiofilm therapy. Moreover, *C. albicans* filamentation induced by different cues that mimics different infectious set-up was significantly hindered. SPB00525 and HTS06170 conferred significant fungal-invasion protective activity to both human enterocytes and *Galleria* larvae cells. This indicates that aryl-carbohydrazides could act as antivirulence agents; however, we cannot, exclude the possibility that SPB00525 or HTS06170 might compromise other virulence traits such as secretion of lytic enzymes or the cytolytic toxin Candidalysin.

In this study, we employed state-of-the-art chemogenomic approaches to elucidate the mechanism of action of the SPB00525 in the opportunistic and model yeast *C. albicans*. The chemogenetic profile of SPB00525 was notably complex in the SC synthetic medium, with numerous mutants associated with various biological processes showing differential abundance compared to the rich medium YPD. As reflected in our RNA-seq data, this complexity may be attributed to the inhibition of Ole1, which leads to significant metabolomic reprogramming to accommodate cellular needs in response to depleted unsaturated fatty acids. Consequently, this creates metabolic vulnerabilities, as evidenced by the sensitivity of a large number of mutants in the minimalist SC medium compared to YPD. This could be also attributed to the fact that, in contrast to the SC medium, YPD contains different species of unsaturated fatty that might buffer SPB00525-mediated Ole1 inhibition. Overall, this underscores the importance of implementing alternative growth media for chemical profiling to narrow down targets of small molecules that impact fungal metabolism.

According to the chemogenetic profiling of SPB00525, our findings suggest that Ole1 may be its target. The significant decrease in various species of unsaturated fatty acids induced by SPB00525, along with the alleviation of its antifungal activity by supplementation with a mixture of palmitoleic and oleic acids, supports our postulate. Therefore, our data align with previous investigations proposing Ole1 as an antifungal target, due to the fact that *OLE1* is an essential gene and it is required for the virulence of *C. albicans* (Krishnamurthy et al., 2004; Becker et al., 2010). Furthermore, various potential Ole1 inhibitors that are chemically distinct from SPB00525 demonstrated potent *in vitro* antifungal activity (Xu et al., 2009; Hoepfner et al., 2014). Two 1,2,4-triazolidine-3-thione analogs (ECC145 and ECC188) (Xu et al., 2009) and a dihydropyridazinone derivative (CMB1257) (Hoepfner et al., 2014) were found to act on Ole1 and exhibited potent activity against *C. albicans* and the model yeast *Saccharomyces cerevisiae*, respectively. Recently, target-based whole-cell screening assay against *C. albicans* Ole1, identified different *in vitro* active molecules that share a similar aryl-carbohydrazide scaffold to SPB00525 and HTS06170 (DeJarnette et al., 2021). Together, our work endorses the antifungal activity of aryl-carbohydrazides and the druggability of their corresponding Δ(9) fatty acid desaturase Ole1 target (Iyer et al., 2023; Vishwakarma et al., 2024). In addition to *ole1*, our chemogenetic profiling uncovered the heterozygous mutant of an uncharacterized gene orf19.5258 as hypersensitive to SPB00525 in both YPD and SC media. Orf19.5258 was annotated as a Pleckstrin Homology (PH) domain containing protein with no additional known functional domain. As PH domain is known to bind specific phosphoinositides in membranes of organelles (Harlan et al., 1994), Orf19.5258 might have a membrane-related function that might depend on the membrane content on UFA and SFA that is dictated by Ole1 activity.

RNA-seq profiling of *C. albicans* and *C. auris* cells underscored many metabolic processes being impacted by early exposure to SPB00525 (15 min). Biosynthetic genes of different classes of membrane lipids including ergosterol, sphingolipids and phospholipids were significantly upregulated suggesting readjustment of lipid metabolism in response to the depletion of unsaturated fatty acids. A similar transcriptional pattern was also reported in human cell lines challenged with the Scd1 inhibitor A939572 (Zheng et al., 2021). In mammalian cells, a disequilibrium in UFA and SFA ratio due to the modulation of Scd1 activity was shown to affect different processes linked to cell growth, differentiation, tumorigenesis and response to external stimuli (Paton and Ntambi, 2009; Oatman et al., 2021; Sen et al., 2023). Intriguingly, we found that in *C. albicans*, the transcriptional profiles of Ole1 inhibition by SPB00525 was similar to the one that characterize cells experiencing hypoxia. Thus, as its human homologs, and through the modulation of UFA: SFA ratio, Ole1 activity might be an important player in modulating fundamental processes such as growth and response to stress. Depletion of oxygen-dependent metabolites are thought to be a mechanism by which fungi sense hypoxia (Ernst and Tielker, 2009). As *Ole1* require oxygen for catalysis, depletion of UFA in response to hypoxia (Burgain et al., 2020) might serve as a cue that drive cellular adaptive mechanisms to tune fungal growth under oxygen-limiting environments. Future investigations are needed to mechanistically probe the role of Ole1 in hypoxia sensing and adaptation in fungi.

## Data Availability

RNA-seq and Barcode-sequencing data have been submitted to the GEO database (https://www.ncbi.nlm.nih.gov/geo/) under accession number GSE267057.

## Supplementary material

Supplementary Figure 1 Transcriptomic analysis of *C. auris* response to SPB00525. GO enrichment of upregulated **(A)** and downregulated **(B)** transcripts of *C. auris* cells exposed to 6 µg/ml SPB00525 for 15 and 60 min.

Supplementary Figure 2 Impact of SPB0052 on ER structure. Sec61-GFP fusion was used as an ER marker to assess the effect of SPB0052 on the structure of *C. albicans* ER. Both cortical and nuclear ERs were labeled with no apparent structural perturbation when cells were exposed to SPB0052. Bar, 10 µm.

Supplementary Figure 3 GSEA graphs of significant correlations between the SPB00525-modulated transcriptome and genes differentially expressed in *C. albicans* under hypoxia. The number below each graph indicates the corresponding transcriptional profile described in **Figure 2I**. The green curve represents the NES curve, which is the running sum of the weighted enrichment score obtained from GSEA software.
