# Supplementary figures and images for: Small molecule inhibitors of fungal Δ(9) fatty acid desaturase as antifungal agents against Candida auris
Source: Front Cell Infect Microbiol. 2024 Aug 30;14:1434939. doi: 10.3389/fcimb.2024.1434939 (PMC11392922; doi:10.3389/fcimb.2024.1434939)

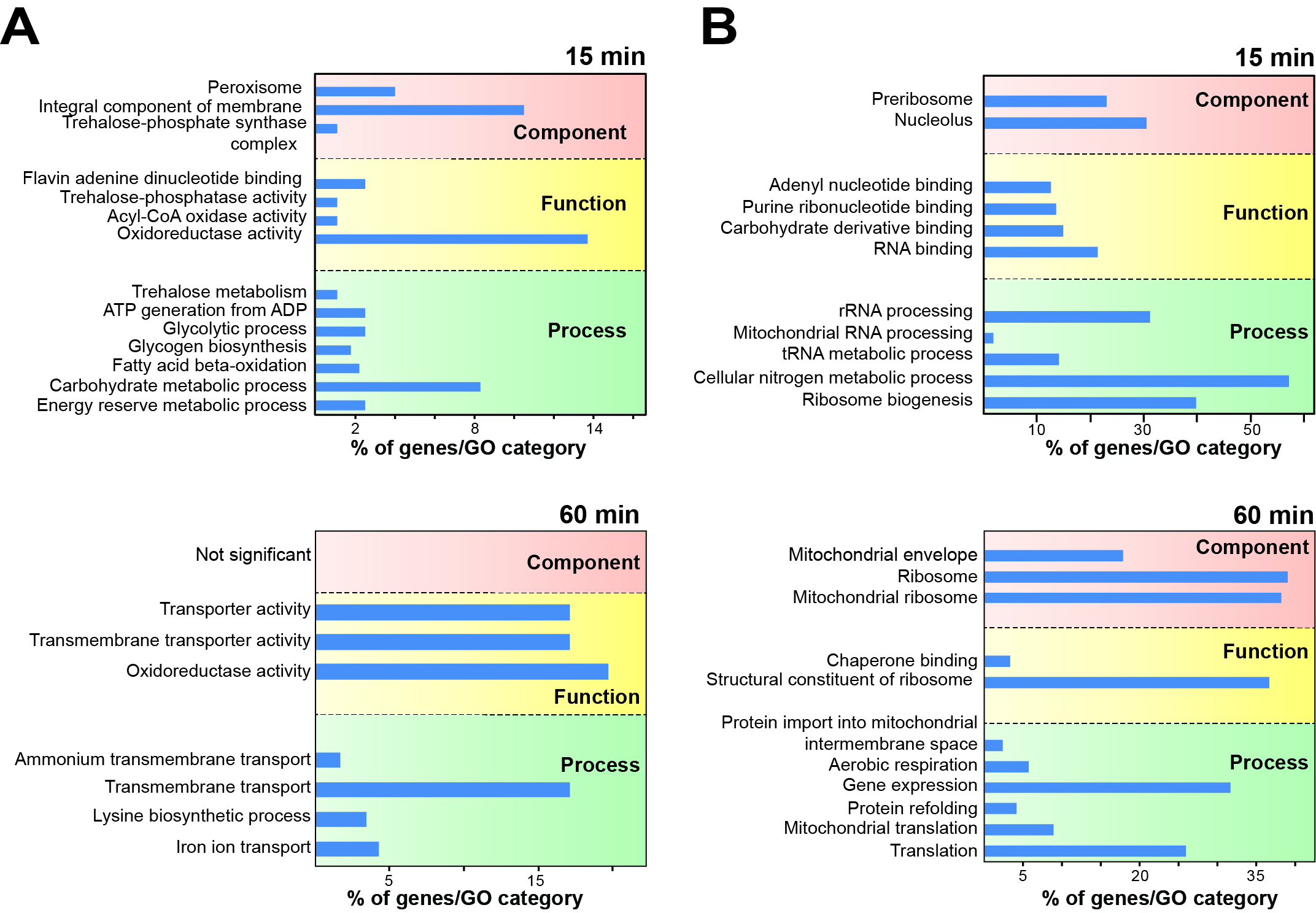

Supplement: Supplementary Figure 1 — Transcriptomic analysis of C. auris response to SPB00525. GO enrichment of upregulated (A) and downregulated (B) transcripts of C. auris cells exposed to 6 µg/ml SPB00525 for 15 and 60 min. [file Image1.tif]

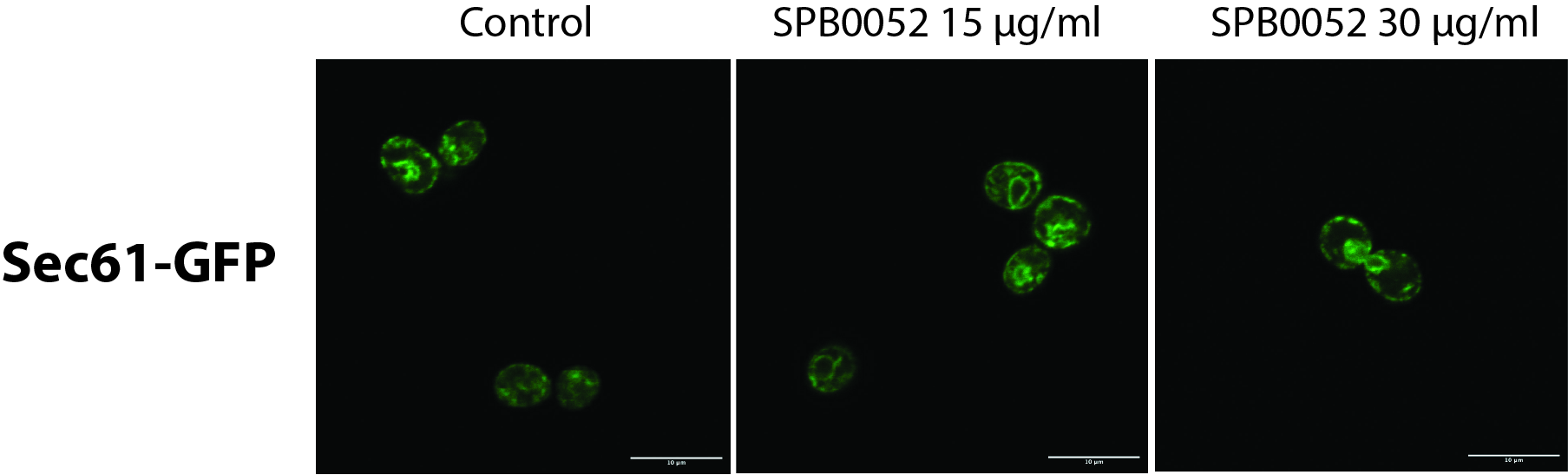

Supplement: Supplementary Figure 2 — Impact of SPB0052 on ER structure. Sec61-GFP fusion was used as an ER marker to assess the effect of SPB0052 on the structure of C. albicans ER. Both cortical and nuclear ERs were labeled with no apparent structural perturbation when cells were exposed to SPB0052. Bar, 10 µm. [file Image2.tif]

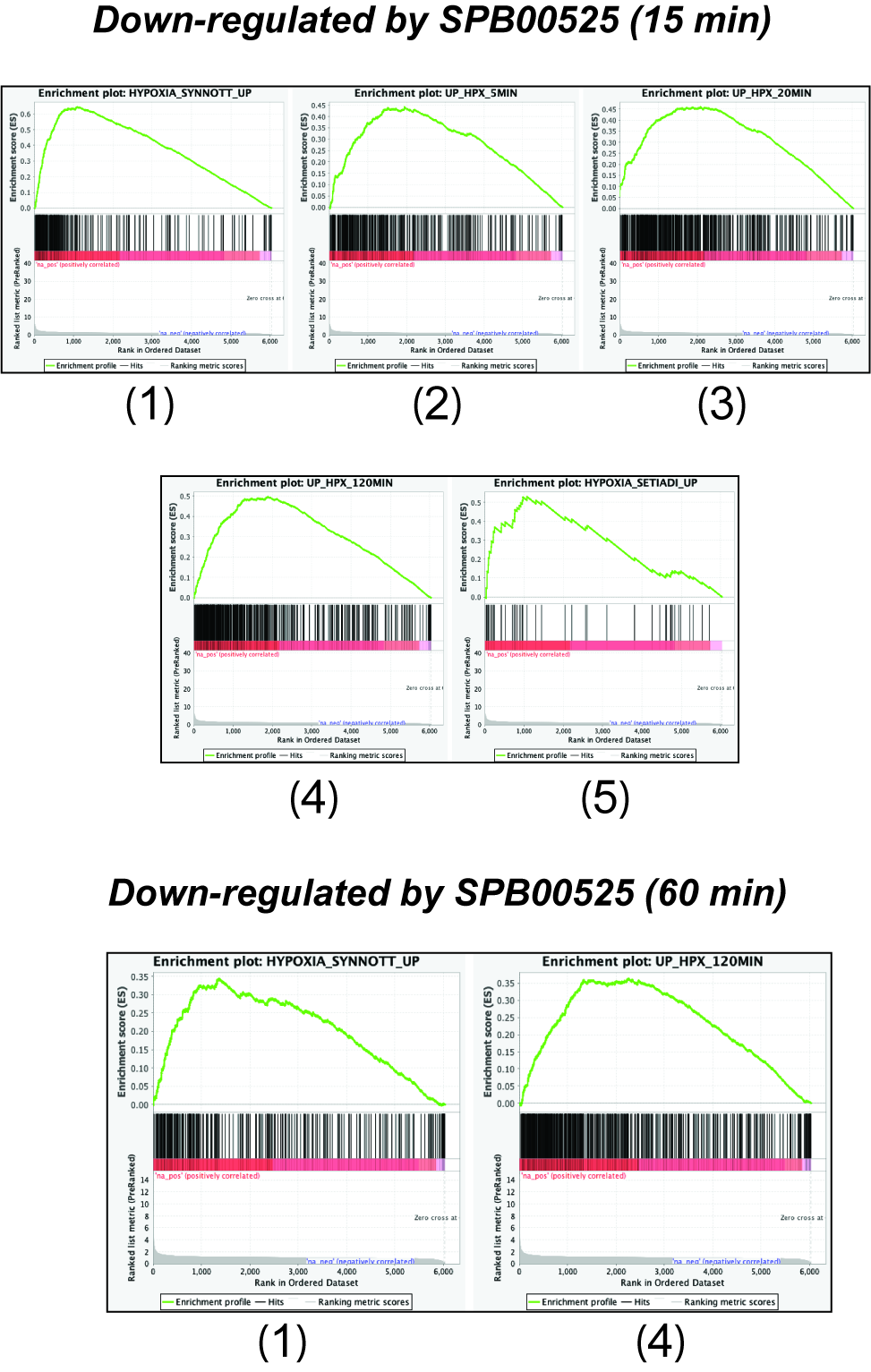

Supplement: Supplementary Figure 3 — GSEA graphs of significant correlations between the SPB00525-modulated transcriptome and genes differentially expressed in C. albicans under hypoxia. The number below each graph indicates the corresponding transcriptional profile described in Figure 2I . The green curve represents the NES curve, which is the running sum of the weighted enrichment score obtained from GSEA software. [file Image3.tif]
